# Enhancing quality assurance in forensic medicolegal opinions: A standardized training and auditing framework for Arab countries

**DOI:** 10.1016/j.fsisyn.2025.100655

**Published:** 2026-01-06

**Authors:** Mamdouh Kamal Zaki, Zahraa Khalifa Sobh

**Affiliations:** a Senior Consultant of Forensic Medicine, Egypt; b Forensic Medicine and Clinical Toxicology Department, Faculty of Medicine, Alexandria University, Egypt

**Keywords:** Quality assurance, Forensic medicolegal opinion, Standardized training, Auditing, Arab countries

## Abstract

Quality assurance in medicolegal (ML) reporting is essential to ensure accuracy, consistency, and compliance with legal and scientific standards. Since forensic opinions significantly influence judicial decisions, it is crucial to uphold high standards. In the Arab region, there are notable differences in forensic medicine practices among countries, emphasizing the urgent need for unified standards. Therefore, this initiative aims to improve the quality of ML reporting by proposing a standardized training and auditing framework based on international best practices, tailored to the realities of Arab forensic institutions. A key part of this article is developing a training framework grounded in best practices to unify forensic medical examiner competencies and reduce variability. At the same time, a specialized auditing framework has been created for senior forensic leaders to improve institutional accountability. This includes implementing a structured auditing protocol that uses newly adopted standardized checklists to assess report quality and detect non-conformities. Additionally, institutional leaders are encouraged to systematically investigate the root causes of substandard opinions within their organizations using Root Cause Analysis (RCA) methodology. Identifying these root causes offers opportunities for improvement. We recommend implementing this initiative through a Training of Trainers (TOT) model. This method enables institutional leaders and senior practitioners to disseminate standardized practices across all levels effectively. The multiplying effects of this initiative are vital for enhancing quality assurance and promoting continuous improvement within forensic practice across Arab countries.

## 1 Background

Forensic medicolegal (ML) opinions significantly contribute to the administration of justice. Indeed, ML opinion formulation is complicated by various factors. The forensic medical examiners' idiosyncrasies, speculations, and false intuitions compromise the ML opinion [1]. Also, the involvement of forensic medical examiners with varying levels of competency in managing ML cases can lead to inconsistent quality in ML reports. Case complexity is another challenge that could affect report quality [2]. In addition, factors related to legal proceedings can also affect the quality of reports. Inadequate police reports and inaccurate medical records can impede the formation of a well-supported opinion [3]. Furthermore, language barriers can undermine the reliability of results, especially when translators lack a medical background. Limited resources in the ML institute might affect reports as well [4]. Thus, across Arab countries, wide variations are observed in ML reports in forensic medicine practice, attributed to disparities in educational backgrounds and the lack of standardized guidelines across institutions [5].

It is worth mentioning that the responsibilities of personnel engaged in forensic medicine practice differ significantly between Arab and Western countries. In many Arab nations, forensic medical examiners examine both living and deceased individuals for ML purposes. In contrast, Western countries often adopt a more specialized approach [6]. Additionally, forensic medicine across Arab countries is shaped by unique cultural and religious considerations, leading to practices that differ from those in Western nations. Regional considerations regarding cultural views on autopsy procedures influence the autopsy rate and the extent of postmortem examinations [7,8].

Despite the availability of international guidelines, many ML institutions in the Arab region lack structured frameworks for training and auditing, leading to variability in report quality, examiner competence, and institutional accountability [5]. Therefore, the current initiative aims to improve standardized forensic practices, particularly in opinion formulation in ML reports requested by legal authorities in Arab countries, through two interconnected components: (1) adapting a training framework based on internationally recognized best practices to unify core competencies and minimize variability among examiners; and (2) implementing a structured auditing protocol to enable systematic evaluation of ML reports. The auditing process incorporates Root Cause Analysis (RCA) to identify factors behind substandard ML opinion. This initiative is firmly rooted in the best international practices established by recognized institutes in forensic medicine [[9], [10], [11], [12], [13], [14], [15], [16]]. Along with the international guidelines, this initiative was guided by the authors' extensive field experience.

## 2 Training framework for optimum opinion formulation in ML reports

**Fig. 1:**
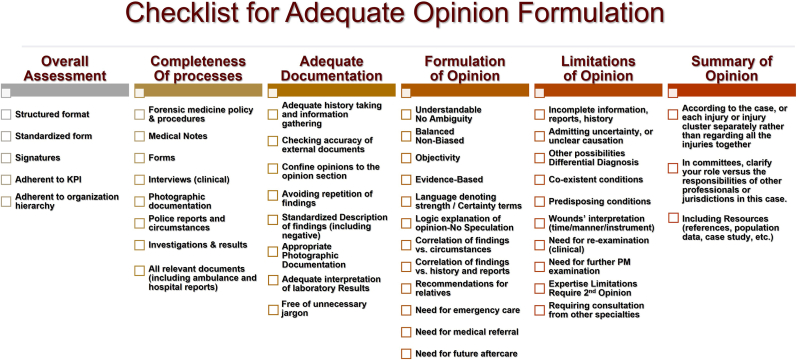
Checklist for adequate formulation of forensic medicolegal opinions.

A quality-based training framework was adopted, incorporating a standardized checklist to guide the structured formulation of ML opinions (Fig. 1). Accurate ML opinions depend on thorough processes beforehand. Therefore, the checklist is based on best practices outlined in forensic medicine textbooks on legal proceedings [17], clinical forensic medicine [18], and forensic pathology, as well as related autopsy procedures [19,20]. The checklist provided could guide forensic medicine practitioners in formulating ML opinions. It also serves as the foundation for a Training of Trainers (TOT) strategy that ensures standardized training is applied properly across different institutes. This tool standardizes key reporting elements, and the steps are as follows:

- Overall assessment:

The administrative aspects of the ML report must be carefully considered. The report must be organized in a structured format, beginning with the circumstances and relevant history. This should be followed by a detailed forensic medical assessment, concluding with an opinion. Each section should be presented in a logical sequence [9].

Each medical authority adopts standardized forms to ensure completeness and organization, which may include printed templates or electronic forms on a secure platform. Reports must adhere to the institute's quality standards for completeness and competency. Relevant key performance indicators (KPIs) should be utilized to ensure that reports meet these quality requirements [21]. All forensic medical examiners managing the case must sign the ML report. Signatures can be handwritten or electronic. The reports must also be authenticated with a stamp from the issuing authority in alignment with the organization's hierarchy.

- Completeness of processes:

Thoroughness in forensic medicine processes ensures the professionalism and credibility of the report. The proper ML assessment consists of a series of procedures, each of which may include several steps. For example, an adequate external examination of ML cases involves inspecting clothing, examining the undressed body, documenting photographs, performing radiological investigations, and conducting toxicological analyses. Notably, the forensic medical examination process is customized for each case [9,14,22,23].

Police investigations should be thorough and timely. Having sufficient information about the circumstances before ML examination aids examiners in reaching accurate conclusions. Timing is critical in ML assessments; delays can significantly undermine their value [24]. For instance, trace evidence can be lost in sexual assault cases, wounds may heal, and decomposition can obscure essential findings. Therefore, in certain situations, forensic medical examiners may need to conduct their examinations quickly, even before receiving all relevant data from legal authorities. In such cases, a comprehensive and meticulous examination should be performed, and all available samples must be collected for further investigation [16]. Forensic medical examiners should formally request the missing data from the police to formulate a sound ML opinion.

In live cases, interviews or interrogations must adhere to the ML institute's policies and procedures. Photographic documentation of all body parts should be conducted in both living and deceased cases. Both positive and negative findings are photographed in accordance with the standards in this context. It is essential to note that photographic documentation requires separate consent [25]. In addition to ML examination, investigations are crucial in case management. Each case requires specific investigations that provide vital information to help resolve legal disputes. For laboratory analyses, it is essential to sample, store, and analyze samples in accordance with the policies and procedures of ML institutes that comply with international standards [10].

Furthermore, radiological investigations should be customized to the specifics of each case. For example, routine radiological investigations may only require X-ray scans of the body's core in most scenarios. However, in cases of road traffic accidents or firearm injuries, full-body scans are necessary. Additional views from plain X-rays may also be requested. Modern imaging modalities could be applied whenever required [26].

Medical notes prepared by forensic medical examiners during case management play a crucial role in enhancing the completeness of ML assessments. These unofficial documents may be stored on electronic platforms, serving as valuable backups for essential data that could assist in auditing and protecting forensic medicine examiners from liability in legal cases.

In addition to maintaining medical notes, forensic medical examiners must complete and retain official ML forms, including registration, evidence collection, referral, consent, and request forms. Furthermore, they must collect and preserve all relevant documents, such as hospital and ambulance records [27].

- Adequate documentation:

All steps involved in formulating ML opinions must be documented in detail. Accurate documentation starts with thorough history taking and information gathering. It is crucial to verify the accuracy of any external documents. The ML findings should be described comprehensively and supported by appropriate photographic documentation. Furthermore, the results of laboratory and radiological investigations need to be interpreted accurately [9,16]. The ML documentation for these steps must adhere to established quality standards. The opinion should be confined to the opinion section of the report. Report writers should avoid redundancy and repetition of findings. Additionally, the report must be free of unnecessary jargon that could confuse legal and judicial authorities [28].

- Formulation of opinion

The opinion connects various factors, such as circumstances, medical history, and death-scene investigations, to provide a logical, reasoned explanation. Therefore, ML opinion must be based on evidence, free from bias, and presented objectively. In some instances, it is necessary to include scientific facts to help readers understand the scientific information supporting ML opinion [28].

The ML opinion should be clearly articulated in understandable language, avoiding ambiguity. It should convey the strength or certainty of the conclusions drawn [29]. Writing ML reports in Arabic is of utmost importance in Arabic-speaking countries. This practice ensures clarity, accuracy, and reliability. Utilizing the native language facilitates effective communication between forensic medical examiners and legal authorities, reducing the likelihood of misunderstandings and enhancing the credibility of the reports in court [30]. ML reports must be translated into other languages in cross-border investigations and proceedings involving non-Arabic-speaking stakeholders. In these instances, professional translation is vital to maintain the integrity of the original report. Accurate translation, using standardized medical and legal terminology, is essential for conveying the intended meaning. Inadequately executed translations could mislead justice by omitting crucial details or misinterpreting evidence [31]. Therefore, it is vital to engage professional translators who are well-versed in medical and legal terminology to ensure the effectiveness of international legal communication.

It is essential to understand that the primary purpose of ML opinion is to serve justice. However, in some instances, the conclusion may include recommendations for the individual being examined, such as the need for emergency care, medical referrals, or future aftercare. Additionally, reports may consist of recommendations for family members and healthcare authorities, especially in cases involving infectious diseases.

- Limitations of opinion:

Forensic medical examiners should honestly mention limitations of the given opinion, such as incomplete information, unclear causation, possible alternative diagnoses, and coexisting or predisposing conditions. Acknowledging uncertainty and clarifying the examiner's role and responsibilities in managing the case are also essential. The need for future examination in living cases and further postmortem investigations should be mentioned. Experience limitations should be clearly stated, and consultation should be sought in challenging cases [32,33].

- Summary of opinion:

This section is the most crucial part of ML reports, as it is the primary focus for legal and judicial authorities. It concisely illustrates the ML opinion, explicitly addressing the causes and manner of injuries or death. The summary responds to the questions posed by the legal authorities who requested the ML report. Each injury or group of injuries must be discussed individually and briefly. Report writers could refer to the scientific background mentioned earlier in the report. Comments on significant and pertinent alternative findings that could explain the observed results are also provided.

## 3 Framework to empower auditing and RCA of substandard ML reports

A targeted framework is adopted to hold forensic medicine leaders accountable and to conduct quality audits of ML reports in Arab countries. A structured auditing protocol, anchored by standardized checklists, has been developed to systematically assess report quality, ensure compliance with best practices, and identify non-conformities. Additionally, implementing RCA is proposed as an effective methodology to uncover factors contributing to substandard opinion formulation within ML institutions. This integrated approach enables senior professionals to spearhead institutional quality improvement.

### 3.1 Auditing processes of non-conformity in ML opinions

The auditing process ensures the reliability of ML opinions in forensic reports. The conducting of systematized assessments is essential to guarantee that auditing can identify gaps, reduce errors, and uphold the integrity of expert opinions [34]. A forensic medicine auditor is a qualified medical professional trained in forensic medicine who evaluates the quality and compliance of ML procedures. Essential qualifications include postgraduate training, legal and ethical knowledge, practical casework experience, and familiarity with quality assurance standards [35]. The auditor must remain objective, confidential, and independent, utilizing strong analytical and communication skills to identify deficiencies and recommend improvements, thereby ensuring the integrity of forensic services [36].

**Fig. 2:**
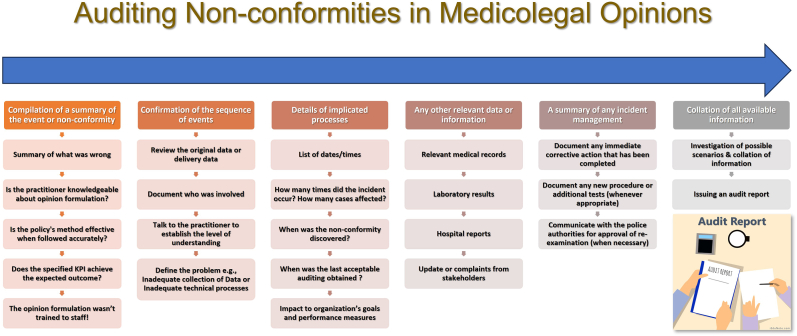
Auditing processes of non-conformity in forensic medicolegal opinions.

To effectively address non-conformity in ML, we recommend following a structured approach that ensures thorough auditing. Fig. 2 illustrates a structured flowchart outlining non-conformity auditing processes in ML opinions. The provided approach for auditing the non-conformity process in ML opinions was formulated in compliance with the literature of quality assurance in forensic medicine practice [[37], [38], [39], [40], [41], [42]].

The auditing process begins with a summary of the causes behind the non-conformity. Assessing how well policies and procedures were implemented and whether KPIs were met is essential. Additionally, the report writers' knowledge of opinion formulation should be evaluated, as should the adequacy of staff training. Following this, the sequence of events is confirmed. All original data and records should be carefully reviewed. All involved individuals should be nominated and discussed to determine at which stage of management the non-conformity took place. Then, the affected processes are analyzed, including recording dates and times, determining how often the incident happens, noting when the non-conformity was detected, and assessing its impact on ML opinions, organizational goals, and performance metrics. Next, any other relevant data should be collected, such as medical records, laboratory results, and stakeholder updates. It is crucial to summarize the incident management, including immediate corrective actions. Additionally, any required procedures or tests should be documented. Police authorities might need to be contacted for approval if reexaminations are necessary. The final step includes gathering all available information and exploring possible scenarios. In the end, a comprehensive audit report is generated.

To formulate a checklist for auditing ML reports in forensic medicine, we adopted a structured format inspired by the Accreditation Standards Checklist of the National Association of Medical Examiners (NAME) [12,21]. This approach ensures objectivity, promotes evaluation consistency, and supports systematic audits. Each audit item is uniquely numbered and worded to elicit a definitive response of “Yes,” “No,” or “N/A" (not applicable). “No” response indicates a deficiency, which is then categorized based on its impact on ML reporting. Phase I deficiencies are non-critical deviations that, although less than ideal, are unlikely to affect report quality directly. Meanwhile, Phase II deficiencies indicate breaches of essential standards that could significantly affect the integrity and reliability of the ML report (Table 1).

**Table (1):** Template for auditing process for the ML report.

Section		Criterion	Phase (І/ІІ)	Results (Y, N, NA)	Comment
**1**		**Summary of Event or Non-Conformity**			
	**a**	Clarity of documentation	**ІІ**		
	**b**	Effectiveness of policy adherence	**І**		
	**c**	Practitioner's expertise	**І**		

**2**		**Sequence of Events Confirmation**			
	**a**	Completeness of records	**ІІ**		
	**b**	Involvement accuracy	**І**		
	**c**	Problem identification	**ІІ**		

3		**Details of Implicated Processes**			
	**a**	Timeliness of incident detection	**ІІ**		
	**b**	Scope of impact	**ІІ**		
	**c**	Auditing history	**І**		

4		**Relevant Data Inclusion**			
	**a**	Availability of medical records	**ІІ**		
	**b**	Lab results inclusion	**ІІ**		
	**c**	External reports	**ІІ**		

5		**Incident Management Summary**			
	**a**	Corrective actions taken	**ІІ**		
	**b**	Communication with authorities	**ІІ**		
	**c**	New protocols applied	**І**		

6		**Collation of Information**			
	**a**	Thoroughness of analysis	**ІІ**		
	**b**	Opinion completeness	**ІІ**		

Y: Yes.

N: No.

NA: Not Applicable.

**Phase I deficiency:** minor deviation that is less than ideal but unlikely to impact report quality directly.

**Phase II deficiency:** violations of a critical standard that could substantially affect the integrity and reliability of the ML report.

### 3.2 Root Cause Analysis (RCA) methodology

Root cause analysis is a well-established quality tool used to identify and categorize potential causes of a problem [43]. RCA is a fundamental element of problem-solving in quality management across various fields, including forensic medicine [28,37]. The RCA process starts by defining the undesired outcome, which in this study was erroneous or substandard opinions in ML reports. Then, we meticulously collect all relevant data. Next, we thoroughly investigate all factors that could contribute to the undesired outcome. The identified root causes were then categorized into six main headings: personnel, procedures, measurement, environment, equipment, and materials [37,44]. In this article, the causes of erroneous and substandard ML reports were identified following a comprehensive review of the relevant literature [22,23,[45], [46], [47], [48]].

Table 2 illustrates the classified root causes of erroneous/substandard ML reporting. It discusses how each root cause could impact the quality of ML opinion. These causes were categorized as follows:

- Personnel

**Table (2):** Root cause analysis of erroneous/substandard opinion in medicolegal reports.

Category	Root Causes	Potential Impact
**Personnel**	- Defective knowledge/experience for ML opinion formulation	Low quality of reports
	- No official unified training programs for practitioners	Lack of knowledge - poor performance - inconsistent practices
	- Unclear responsibilities in report formulation & auditors	Presence of gaps in responsibility - duplication of efforts.
	- Increased workload and limited human resources	Burnout - omission of important details -delay in issuing reports
	- Resistance of leaders to improvement	Persistence of pitfalls - hindering improvement actions.

**Procedures**	- Defective/no P&P for ML reporting	Heterogeneity in reports
	- Lack/no communication with stakeholders	No collaboration - Misunderstandings of data - delays ML proceedings.
	- No implemented policy for photography	Substandard photographic documentation - undermined reliability

**Measurements**	- Inefficient electronic system for ML reporting	Hinder stakeholders' collaboration - delays in ML proceedings
	- Inadequate/no internal/external auditing	Lack of oversight - low quality of reports
	- Unreliable KPIs	Inadequate performance assessment - inability to track progress
	- Restriction/no corrective actions	Failure to identify errors - repetition of errors

**Environment**	- No adequate place for ML examination and handling evidence	Loss of evidence - inadequate workflow
	- Security issues and a stressful work environment	Increase the probability of errors

**Equipment**	- Substandard photographic equipment	Poor image quality - difficulty in examining findings
	- Inadequate illumination devices	Difficulty inspection - inadequate assessment
	- Shortage of computer devices	Delay in workflow - reduced efficiency
	- Poor internet connections	Limitations in accessing information - delayed communication

**Materials**	- Inappropriate materials for sampling/packaging	Compromising the integrity of evidence
	- Shortage of medical supplies	Hindering the process of ML examination
	- Shortage of materials required for reporting	Hindering appropriate documentation

Numerous personnel-related flaws contributed to poor ML opinions. The main issue is insufficient knowledge and experience among those managing ML cases. This is compounded by the absence of official, unified training programs, leaving forensic medical examiners without standardized guidance [49]. Additionally, unclear delineation of responsibilities among those involved in report preparation and those auditing them could result in inconsistent practices, repetition of work, and poor oversight [50]. Excessive workloads and insufficient staffing place additional pressure on forensic medical examiners, increasing the probability of errors and undermining the quality of reports [51]. Lastly, resistance from leadership to adopt improvements hinders progress toward best practices.

- Procedures

Absence of clear policies and procedures (P&P) for ML reporting results in inconsistencies in report preparation [28]. Furthermore, a lack of communication with stakeholders, such as legal authorities or forensic medicine experts, leads to incomplete or inconsistent data in the reports [52]. The absence of a formal photography policy might also undermine the accuracy of the evidence.

- Measurement

Inefficient electronic systems for ML reporting hinder stakeholder collaboration and time-efficient data processing [53]. Additionally, inadequate auditing means an absence of oversight and accountability. This allows errors to go unnoticed and promotes their recurrence. Unreliable KPIs lead to insufficient performance assessment and complicate progress tracking [54]. Lastly, the restriction or absence of corrective actions hinders continuous improvement, allowing errors to persist without intervention.

- Environment

The lack of a proper space for examining cases could hinder accurate assessment. Additionally, the absence of a dedicated area for handling and storing evidence leads to poor storage, handling, or analysis. Security concerns might also expose sensitive data to breaches. A stressful work environment negatively affects forensic medical examiners' performance [55].

- Equipment

Poor photographic equipment reduces the clarity and detail of captured photos, which are essential for evidence documentation. Additionally, inadequate lighting devices further diminish the quality of photographic documentation, resulting in low-quality images that are hard to interpret [28]. A shortage of computer devices may also delay report issuance and reduce data processing efficiency. Poor internet connectivity further impedes communication, access to vital data resources, and the timely sharing of reports.

- Materials

Using inappropriate materials for sampling and packaging can lead to the mishandling of evidence, compromising its reliability. A shortage of medical supplies might hinder proper examination. Similarly, a lack of materials needed for reporting, including forms and documentation tools, could affect report preparation. In forensic medicine practice, the identified defects highlight opportunities for improvement [37]. The defects and their magnitudes vary greatly across ML institutes. Therefore, we urge the heads of ML institutes to use the provided RCA to identify the sources of erroneous or substandard ML opinions. Once the root causes are determined, specific corrective measures can be implemented to address these defects. Using RCA promotes ongoing improvement in the quality of ML reports, which strengthens their integrity and credibility. However, it is crucial to recognize that contextual differences, such as variations in institutional resources, organizational structures, and trainer preparedness, can challenge the uniform application of TOT and auditing models. Therefore, there is a need for adaptive mechanisms that balance standardization with responsiveness to local conditions. The TOT and auditing models should be customized to address the specific issues identified during RCA, ensuring practical responsiveness.

## 4 Conclusions

This initiative aims to enhance quality assurance in forensic ML opinions within forensic medicine institutes across Arab countries. A comprehensive training framework based on best practices in forensic medicine has been developed to unify core competencies and reduce variability among forensic medical examiners. Additionally, a specialized framework has been provided to auditors to ensure effective accountability. This includes using a structured auditing protocol with standardized checklists to evaluate ML report quality and identify non-conformities. RCA has been implemented to systematically identify the causes of substandard opinions within ML institutions. We recommend delivering this initiative through the TOT model to ensure consistent dissemination and sustainable implementation. Institutional leaders and senior forensic medicine practitioners and consultants are advised to utilize the provided initiative to ensure standardized practices across all levels of ML institutions. This multiplier effect is essential for strengthening a unified culture of quality and accountability, which encourages ongoing improvement in forensic medicine practices across Arab countries.

## CRediT authorship contribution statement

**Mamdouh Kamal Zaki:** Writing – review & editing, Writing – original draft, Visualization, Validation, Supervision, Resources, Methodology, Investigation, Conceptualization. **Zahraa Khalifa Sobh:** Writing – review & editing, Writing – original draft, Visualization, Validation, Methodology, Investigation.

## Ethical considerations

The current study received approval from the research ethics committee of the Faculty of Medicine at Alexandria University (Serial Protocol Number: 0307307, FWA Number: 00018699, IRB Number: 00012098). Informed consent was not necessary.

## Funding

None.

## Declaration of competing interest

The authors declare that they have no known competing financial interests or personal relationships that could have appeared to influence the work reported in this paper.
